# Snack Confusion: Parents perceive baby, child and adult snacks as more similar than they actually are

**DOI:** 10.1016/j.crfs.2025.101113

**Published:** 2025-06-12

**Authors:** Alenica Hässig-Wegmann, Sergio Román, Luisma Sanchez-Siles, Michael Siegrist

**Affiliations:** a ETH Zurich, Department Health Science and Technology (D-HEST), Consumer Behaviour, Switzerland; b Marketing Department, Facultad de Economía y Empresa, Universidad de Murcia, 30100, Murcia, Spain; c Institute for Research and Nutrition, Hero Group, 5600, Lenzburg, Switzerland

**Keywords:** Commercial baby snacks, Evaluations, Food regulatory knowledge, Buying intentions

## Abstract

The consumption of commercial snacks for infants and toddlers has risen significantly, yet limited research has explored how parents evaluate these products across age groups. This study examined parental perceptions of snacks marketed for babies (<36 months), children (commercial snacks with child-appealing packaging), and adults, focusing on attributes such as healthiness, appropriateness, and parental willingness to buy. It also assessed the relationship between parents’ knowledge of the European Union (EU) baby food regulations and their purchasing intentions. An online survey was conducted with 704 parents in Germany. ANOVAs with Tukey post hoc tests were used to compare these measures across snacks for each age group. Additionally, Pearson correlations examined the relationship between food regulatory knowledge and buying intentions. Parents showed minimal differentiation in their evaluations of snack attributes across age groups, despite baby-specific snacks being subject to stricter limits on nutritional composition and contaminants. Our analysis also showed that parents were not fully aware of EU baby food regulations, particularly regarding restrictions on preservatives and pesticides, with 45.5 % and 52.5 % correct answers, respectively. While greater regulatory knowledge was moderately associated with a higher willingness to buy baby-specific snacks, correlations were weak, indicating a knowledge-behavior gap. These findings suggest the need for clearer labeling, stricter regulation of health claims, and educational efforts to help parents distinguish baby-specific food products and make informed choices aligned with stricter regulatory standards. Policymakers can support these efforts by ensuring regulations are effectively communicated and enforced.

## Highlights

• Parents showed minimal differentiation in their evaluation of baby, child, and adult snacks.
• Parents had medium knowledge of EU infant food regulations.
• Regulatory knowledge had minimal influence on willingness to buy baby-specific snacks.
• Clearer labeling and stricter health claims can guide informed snack choices.

## Graphical abstract

**Figure:**
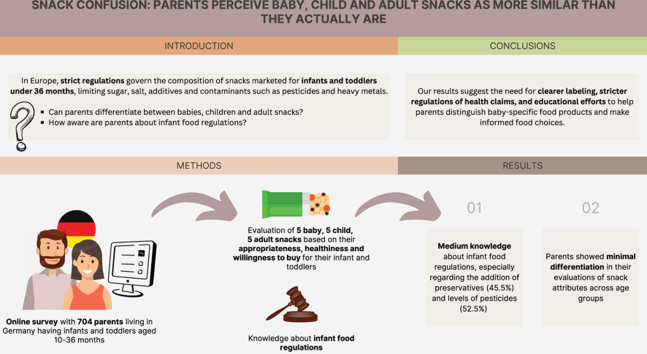

## 1 Introduction

The first 1000 days of life are critical for establishing dietary habits that can persist into adulthood, with significant implications for long-term health (Koletzko et al., 2017). Early exposure to healthy eating patterns can help prevent childhood obesity, which remains a growing public health challenge worldwide (Nicklaus and Remy, 2013; Schwartz et al., 2011). However, dietary practices in infancy and early childhood are shifting, with commercial snacks playing an increasingly prominent role in children's diets. Research has shown that these products often contribute significantly to daily caloric intake, raising concerns about their nutritional quality and potential impact on health outcomes (Deming et al., 2017; Garcia et al., 2020; Shriver et al., 2018). This paper examines how parents of children under 3 years evaluate commercial snacks marketed for babies,^1^ children, and adults, and the role of their food regulatory knowledge in shaping snack purchasing intentions.

### 1.1 The growing role of commercial snacks

Snacks, defined as calorie-containing foods consumed outside regular mealtimes (Boukid et al., 2022; Chaplin and Smith, 2011; Killion et al., 2023), are becoming a substantial part of young children's diets, as global sales have surged over the past decade (Innova Market Insights, 2024.; Statista, 2024). Data from Europe and the U.S. reveal that snacks account for more than a quarter of daily caloric intake for infants and toddlers, with products like crackers, biscuits, pouches, and extruded^2^ snacks driving this trend (Deming et al., 2017; Haszard et al., 2024; Moore et al., 2022; Shriver et al., 2018; Xue et al., 2019).

While these products are convenient and widely available, their suitability for young children has been the subject of growing debate. Some studies recognize that certain commercial baby snacks can serve as a source of nutrients like fiber, vitamins, and minerals (Haszard et al., 2024; Klerks et al., 2022; Swanepoel et al., 2019). However, significant concerns persist regarding their high level of processing, their excessive fat, sugar, and salt content, and their potential to encourage unhealthy eating habits (Dunford et al., 2015; Garcia et al., 2020; Hutchinson et al., 2021; Katiforis et al., 2021; McCann et al., 2022; Pries et al., 2023). These issues are further compounded by marketing practices that make it harder to distinguish between products intended for babies and those for older children or adults. For example, front-of-pack labeling and marketing strategies for non-baby snacks—such as bright colors and cartoon characters—may mislead parents, encouraging them to select less appropriate products for babies (Dixon et al., 2024; García et al., 2019; McCann et al., 2022; Mehta et al., 2012; Mooney and Feeney, 2021). This confusion can lead to the early introduction of snacks with ingredients (e.g., high levels of salt and sugar) and textures, which are not recommended as part of a healthy diet for the vulnerable under-36-month age group. At this stage, their rapid growth and specific developmental needs make infants and toddlers particularly susceptible to inadequate nutrition and harmful contaminants such as pesticides, heavy metals, and toxins (Mehta et al., 2012; Moumin et al., 2020; Roman and Sanchez-Siles, 2025; Word Health Organization, 2019; World Health Organisation, 2019).

### 1.2 Differences across snacks marketed to various age groups

In Europe, strict regulations govern the composition of snacks marketed to infants and toddlers under 36 months, limiting sugar, salt, additives, and contaminants such as pesticides and heavy metals (Commission Directive 2006/125/EC, 2006, Commission Regulation No. 609/2013, 2013 ). These rules aim to protect young children's health and ensure the suitability of these products for their developmental needs. By contrast, snacks for older children and adults are subject to less stringent regulations, allowing for higher levels of sugar, salt, and other additives as well as contaminants (Roman and Sanchez-Siles, 2025).

Despite these protections, recent studies suggest that many commercial baby snacks fail to fully meet regulatory guidelines, raising questions about their nutritional adequacy (Garcia et al., 2020; McCann et al., 2022). Furthermore, differences in nutritional quality between snacks targeted at babies, children, and adults remain a concern. For instance, a recent study found that while baby biscuits generally have higher nutritional quality, products marketed to children (>36 months) often mirror the poor nutritional profiles of adult snacks, underscoring the need for clear distinctions between these categories (Klerks et al., 2023).

### 1.3 Parents’ perceptions of and reasons for using baby snacks

Parents play an important role in shaping their children's dietary habits through their product choices, making it essential to understand how they evaluate and decide on snacks for young children. While snacks play an increasingly important role in infants' diets, studies have only recently begun to explore how parents evaluate snack options. This stream of research has largely relied on qualitative methods (e.g., Damen et al., 2019, 2020a, 2020b; Isaacs et al., 2022; Jacquier et al., 2017; Moore et al., 2021a, Moore et al., 2021b; Reale et al., 2018; Tang et al., 2020; Younginer et al., 2016). A thematic synthesis of 10 qualitative studies found that caregivers viewed some snacks as nutritious while others as less nutritious options. Less nutrient-rich snacks were often highly favored by children but consumed primarily outside the home, requiring some level of restriction. Caregivers commonly used snacks to satisfy hunger or manage behavior, with portions generally described as small (Killion et al., 2023).

Quantitative studies on snacking remain scarce, with most focusing on patterns and parental influences. Hutchinson et al. (2018) reported that 96 % of a sample of 23 Canadian children (mean age = 3.4 years) snacked daily. In the United States, a study of 505 toddlers (1–2 years) by Jacquier et al. (2018) found that milk and milk products were the most common snacks consumed at home, while sweets were most frequently consumed away from home. Surveys of parents have provided additional insights. For instance, Boots et al. (2015), in a study of 611 parents of children aged 2–7 years, found that restrictive feeding practices were linked to greater unhealthy snack consumption. Damen et al. (2019) used food and motivation diaries with 136 Dutch mothers of children aged 2–7 years, identifying healthiness, children's preferences, and established routines as key factors shaping snack choices. In the United States (Moore et al., 2023) found that parental feeding practices had a stronger influence on infant snack consumption than infant characteristics in a sample of 141 parents with babies aged 9–15 months. Finally, an online survey of 271 UK parents with infants aged 4–12 months revealed that vegetable puffs and sticks were the most commonly purchased baby snacks, favored over purees for their convenience, time-saving attributes, and perceived health benefits (Hollinrake et al., 2024).

While these qualitative and quantitative studies offer valuable insights into the factors influencing parents’ snack choices, they often overlook whether parents can distinguish between snacks specifically designed for babies and those intended for older children or adults. This distinction is particularly important, as snacks for children under 3 years are subject to stricter regulations, as described earlier. Understanding whether parents are aware of these regulatory protections and whether such knowledge influences their evaluations and choices is crucial for assessing the effectiveness of these regulations in guiding healthier decisions for this vulnerable age group.

### 1.4 Study objectives

Building on these insights, this study has two key objectives. First, we aim to assess the extent to which German parents’ perceptions of snack attributes—such as appropriateness and healthiness—and their willingness to buy differ across categories of snacks marketed to different age groups: infants and toddlers (<36 months), children (>36 months), and adults. By analyzing both their perceptions of these attributes and their purchasing intentions, we seek to determine whether parents can effectively differentiate between products intended for different age groups and whether existing regulations and marketing practices adequately support healthier and more appropriate snack evaluations and choices among parents of children under 3 years.

Second, we evaluate the relationship between parents' knowledge of baby food regulations and their behavioral intentions to purchase snacks across these age groups. While regulations for baby food are designed to ensure optimal nutrition and protect infants and toddlers from harmful substances, research suggests that parents' purchasing decisions are not always aligned with these aims. This study investigates whether and how regulatory knowledge shapes parents' willingness to buy snacks and whether this awareness helps them make informed regulation-aligned choices. The findings aim to inform policy and educational initiatives to bridge gaps between knowledge and action, promoting healthier and more informed decisions for parents’ choices for their children.

## 2 Material and methods

### 2.1 Study participants

For the present study, the data was collected through an online survey in Germany in October 2024. Respondents were recruited via an internet panel provider (Bilendi & Respondi). Respondents received a small compensation (approximately $8–10) for their participation. Bilendi & Respondi sent the participation link via email to individuals within the target group. Before beginning the survey, participants were required to read and accept an informed consent form, which provided details about the survey's topic, assured confidentiality and anonymity of the data, and outlined participants' rights to withdraw at any time during the survey. Only participants who agreed to the informed consent were able to proceed to the screening questions. To be eligible, participants needed to be the primary caregiver responsible for making dietary decisions and purchasing food for at least one child aged 10–36 months. This age range was selected based on the WHO's definition of complementary feeding, as 10 months mark the period when children begin to consume a greater variety of foods and textures and slowly transition to the regular family diet (World Health Organization, 2023). Furthermore, previous research has shown that from 10 months onward, the consumption of commercial snacks increases in infants' and toddlers' diets (Pries et al., 2016). The upper limit of 36 months was selected due to regulatory considerations, as baby snacks remain subject to stricter regulations up to this age. This study was approved by the ETH Zürich Ethics Commission in October 2024 (EK, 2024-N-301).

After screening, our final sample included 704 participants. Those who did not complete the questionnaire or whose completion times fell below half of the median (to ensure careful responses) were excluded (*n* = 73). Furthermore, two attention-check questions were included to ensure data quality, and all 704 participants successfully answered them, indicating a high level of engagement and attentiveness among the parents in our study. The final sample included 505 women and 199 men, with an average age of 34 years (*SD* = 4). Three percent of the participants (n = 24) had a low education while 43 % (n = 303) had a medium education and 54 % (n = 377) had a high education.^3^ The average age of the respondents’ infants and toddlers was 23 months (*SD* = 3). The larger number of women in our sample reflects the common role of women as primary caregivers, as indicated by the Federal Office of Statistics (Destatis, 2021, p.157, Figure 8). Also, the education distribution is representative of the education in the German population (Federal Office for Education, 2022).

### 2.2 Survey questions

#### 2.2.1 Evaluation of commercial snack product

Participants evaluated 15 different commercial snack products inspired by Klerks et al. (2023). The products were selected from five categories, identified through semi-structured interviews as the most commonly used snack types (Hässig-Wegmann et al., 2025). The five product categories included were: bars (e.g., oat bar), crackers (e.g., oat cracker, pretzel), puffs (e.g., extruded snacks such as vegetable puff), pouches (e.g., fruit-based pouch) and biscuits (e.g., spelt biscuit, butter biscuit). In each category, participants evaluated three products, each of the three products was targeted at a different age group: one snack was designed for babies, one for children, and one for adults. The products were selected from the online stores of the largest infant and toddler food distributors in Germany (e.g., Müller, DM, Rossmann, Aldi, and Lidl). During product selection, efforts were made to ensure that the baby, child, and adult products within each product category were as similar as possible in terms of flavor, texture, and packaging elements, such as marketing claims. For example, if one product featured an organic claim, the other products did as well to maintain consistency in packaging. However, packaging colors varied slightly to avoid creating an unnecessarily difficult task for participants. Instead, our goal was to replicate a realistic shopping scenario that parents might face when choosing commercial snacks for their infants and toddlers. To further ensure a realistic evaluation setting, participants were shown images of products available in actual online stores. Similar to previous studies (Etter et al., 2024; Hässig et al., 2023; Hässig-Wegmann et al., 2024) only the front of each package was displayed, with no ingredient or nutritional information provided. However, the target age for baby snack products was visible to participants on the front of the packaging. Consistent with prior research (Klerks et al., 2023; Lapierre et al., 2017; Mehta et al., 2012), the children's snacks included in our study featured packaging design elements commonly used to attract children. These elements included colorful packaging with multiple or bright colors, promotional characters such as cartoons, and/or unique or playful packaging designs or food shapes.

**Fig. 1:**
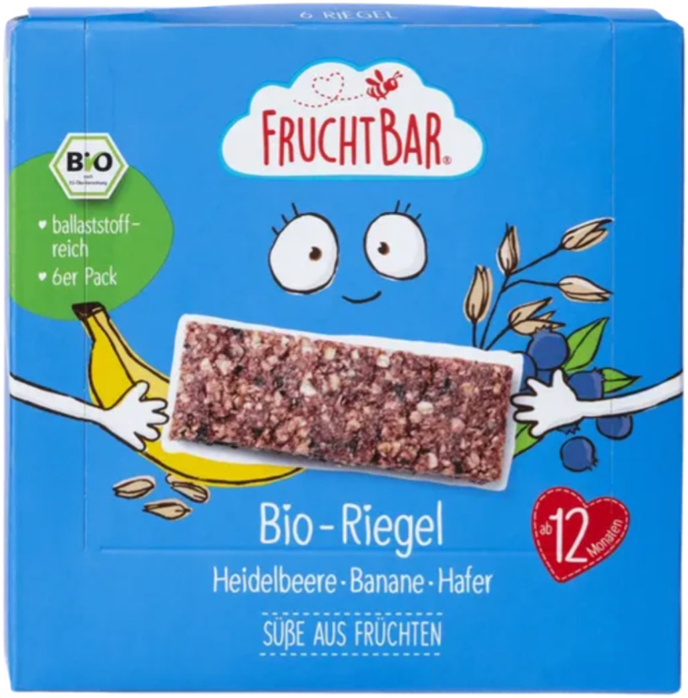
Example of product evaluated by the participants. The following instructions were provided to the participants next to the product image: When responding to the following questions, please think about your child(ren) aged 10–36 months. Try to answer the questions without considering your child's specific taste preferences. *Note*. Information from the German packaging: Organic bar made with blueberries, banana, and oats. Sweetened with fruit. (Green bubble: High in fiber and 6-pack.)

Fig. 1 provides an example of a product shown to participants. For each product, participants evaluated three perception attributes: appropriateness, healthiness, and their willingness to buy. To assess these three attributes the following questions were asked: “How appropriate do you perceive the ingredients of this product for your child?”, “How healthy do you perceive this product for your child?”, and “How likely is it that you would buy this product for your child?”. They were instructed to evaluate each product specifically for their child aged 10–36 months, as some participants had multiple children, not all of them falling within the target age range of this study. Each attribute was rated on a slider scale ranging from 0 (not at all) to 100 (very much) for each product. Participants' ratings for each product on appropriateness, healthiness, and willingness to buy were used for further statistical analysis.

#### 2.2.2 Food regulatory knowledge

To assess participants' knowledge of EU food regulations for infants and toddlers, we adapted the scale developed by Hässig-Wegmann et al. (2024). The scale contained six multiple-choice statements, offering participants the response options of “true”, “false” or “don't know”. The scale included statements regarding permitted ingredients (e.g., salt, sugar, additives) and regulations under EU legislation, such as “Commercial baby foods are allowed to contain colorants” and “EU legislation limits the quantity of salt and sugar in commercial baby food”. All statements, along with the percentages of correct answers, are shown in Table 1. Correct responses by the participants were coded as 1, while incorrect and “don't know” answers were coded as 0. Principal component analysis (PCA) was performed, and the results supported the one-dimensionality of the items. A total sum score of correct answers was calculated as a proxy for participants' knowledge of infant and toddler food regulations, with a maximum possible score of 6 points when answering all questions correctly. The scale had a Cronbach alpha of .63. The sum score was used in for data analysis (*M* = 3.78, *SD* = 1.66).

**Table 1:** Knowledge about baby food regulations and percentages of correct answers.

Item	Correct answers (%)
Commercial baby foods are allowed to contain colorants (false)	n = 434 (61.6 %)
Commercial baby foods are allowed to contain preservatives (false)	n = 320 (45.5 %)
Commercial baby foods are allowed to contain additives (false)	n = 533 (75.7 %)
The regulations for pesticides in baby food are stricter than for conventional foods (correct)	n = 370 (52.5 %)
EU legislation limits the quantity of salt and sugar in commercial baby food (correct)	n = 490 (69.6 %)
EU legislation prohibits the addition of trans fats in commercial baby food (correct)	n = 512 (72.7 %)

### 2.3 Data analysis

The data was analyzed using the statistical programming language R (version 2024.9.0.375; Posit team, 2024).

**Fig. 2:**
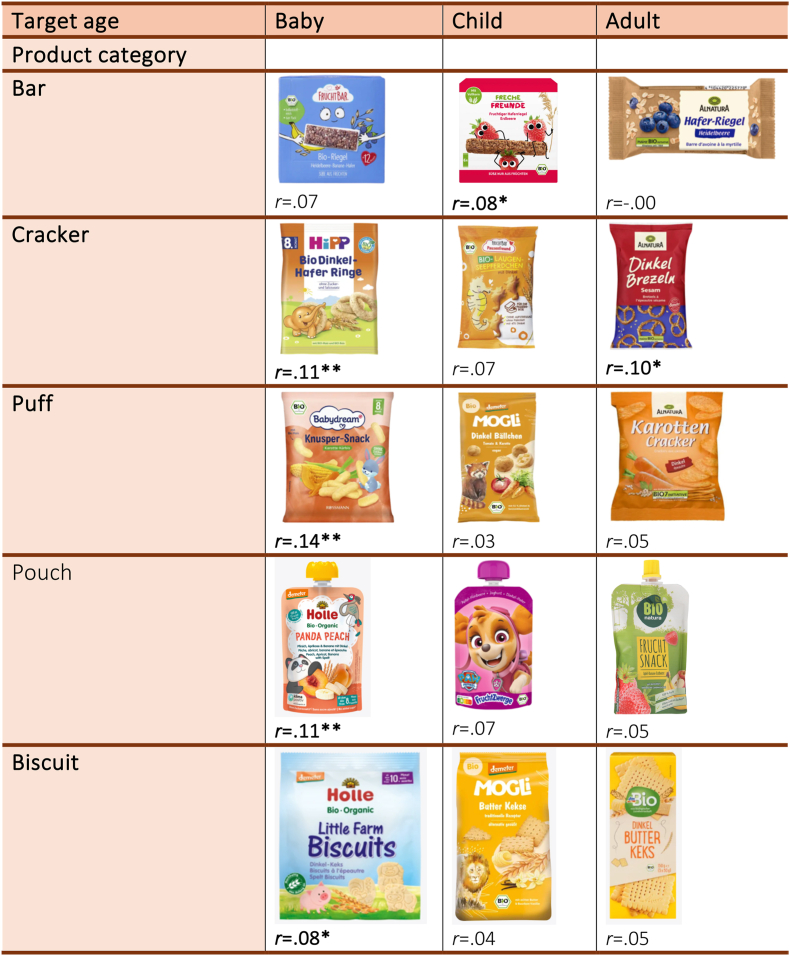
List of products by intended age group and category. Below each product the correlation coefficient (r) is shown between the participants willingness to buy and their knowledge about infant food regulations (N = 704). *Note*: ∗*p* < .05, ∗∗*p* < .01, ∗∗∗*p* < .001. The translations of the information provided on the packaging can be found in Appendix A.

**Fig. 3:**
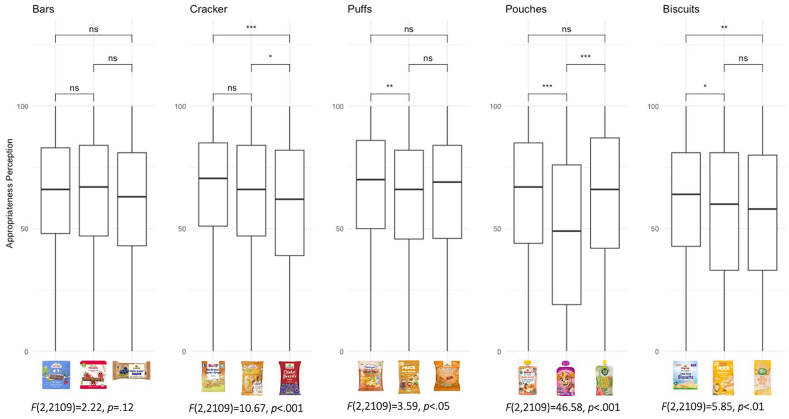
Boxplots of appropriateness perceptions of product types: bar, cracker, puff, pouch and biscuit on a scale from 0 (not al all) to 100 (very much).

**Fig. 4:**
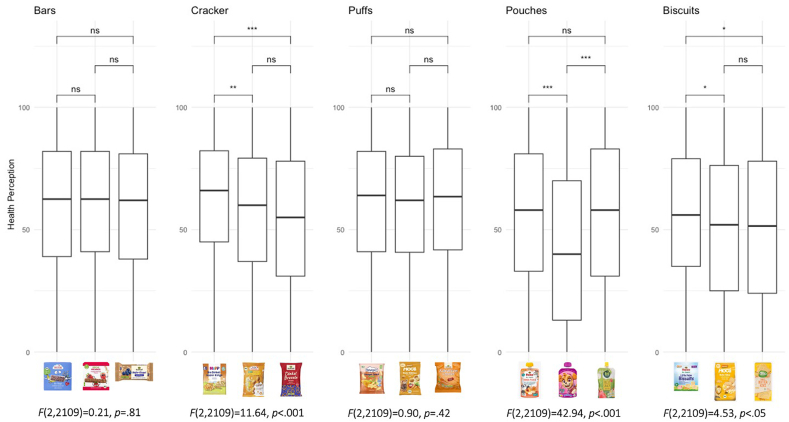
Boxplots of healthiness perceptions of product types: bar, cracker, puff, pouch and biscuit on a scale from 0 (not at all) to 100 (very much).

**Fig. 5:**
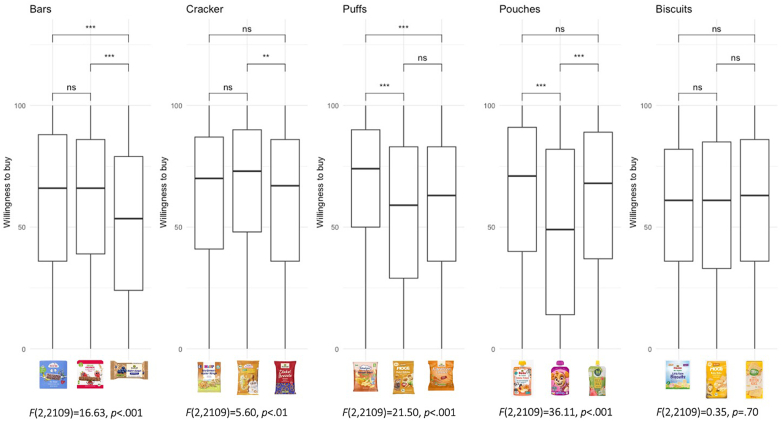
Boxplots of willingness to buy product types: bar, cracker, puff, pouch and biscuit on a scale from 0 (not at all) to 100 (very much).

Mean values and standard deviations were calculated for all 15 products, which included five product categories (e.g., bars, crackers, pouches, puffs, and biscuits) and were divided by three different target ages (e.g., baby, children, adults). Fig. 2 provides an overview of the products used, categorized by product type and target age group. For each product, participants evaluated three perception attributes: appropriateness, healthiness, and willingness to buy. To assess these three attributes the following questions were asked: “How appropriate do you perceive the ingredients of this product for your child?”, “How healthy do you perceive this product for your child?”, and “How likely is it that you would buy this product for your child?”. To illustrate participants’ perceptions regarding the five product categories in terms of appropriateness, healthiness, and willingness to buy, a series of boxplots were generated (Fig. 3, Fig. 4, Fig. 5) using a scale from 0 (not at all) to 100 (very much). To examine whether participant perceptions varied significantly between snacks targeted at babies, children, and adults, ANOVAs were conducted, each followed by a Tukey post hoc test. Finally, Pearson correlations were computed to examine the relationships between food regulatory knowledge and buying intentions (Fig. 2).

## 3 Results

### 3.1 Willingness to buy, perceived appropriateness and healthiness

Participants’ perceptions of the 15 products in terms of appropriateness and healthiness as well as their willingness to buy are shown in Appendix B. Fig. 3, Fig. 4, Fig. 5 present the distribution of responses for appropriateness, healthiness, and willingness to buy across the five product categories, along with the corresponding F-values and Tukey post hoc test results.

#### 3.1.1 Appropriateness perception

For the product category bars, no significant differences were found in parents' perception of the appropriateness of the product to their child. In other words, bars for babies, children, and adults were perceived to be equally appropriate. In the cracker category, there were also no significant differences between products targeted at babies and children, suggesting that for both bars and crackers, parents perceived products aimed at babies and children as similarly appropriate (see Fig. 3). For puffs and biscuits significant differences in appropriateness perception emerged between baby and children's products. However, while parents rated baby puffs (*M* = 65.9, *SE* = .98) slightly higher in appropriateness compared to those for children, these differences were minimal. Similarly, for the biscuits, parents perceived the product targeted at babies (*M* = 60.8, *SE* = .94) as more appropriate to the biscuits targeted at children (*M* = 56.8, *SE* = 1.52, *p* < .05) and adults (*M* = 56.0, *SE* = 1.07, *p* < .05). Significant differences emerged in the pouch category: the pouch aimed at children (*M* = 48.8, *SE* = 1.13) was rated significantly less appropriate than those for babies (*M* = 62.3, *SE* = 1.07, *p* < .001) and adults (*M* = 61.9, *SE* = 1.59, *p* < .001), though no differences were found between the baby and adult pouches.

#### 3.1.2 Healthiness perception

Participants' perceptions of healthiness among snacks for babies, children, and adults were generally similar, with minimal differences across target ages (see Fig. 4). No significant differences were found in healthiness perceptions for the bars and puffs categories. In the pouch category, the children's pouch (*M* = 43.2, *SE* = 1.17) was rated as less healthy than both the baby (*M* = 56.2, *SE* = 1.65, *p* < .001) and adult pouch (*M* = 56.6, *SE* = 1.12, *p* < .001), with no difference between baby and adult pouch. For biscuits, the baby biscuit (*M* = 55.5, *SE* = 1.59) was perceived as slightly healthier than those for children (*M* = 51.5, *SE* = 1.65, *p* < .05) and adults (*M* = 51.3, *SE* = 1.44, *p* < .05), though differences between the healthiness perception were small. Furthermore, some differences appeared in the crackers category, where the baby cracker (*M* = 61.8, *SE* = 1.04) was seen as healthier than the children's (*M* = 57.6, *SE* = 1.45, *p* < .01) and adult versions (*M* = 54.7, *SE* = 1.47, *p* < .001).

#### 3.1.3 Participants willingness to buy

The results for parents' willingness to buy products across target ages suggest that, in three out of five product categories, parents did not show a significantly greater willingness to buy baby products compared to children's products (e.g., bars, crackers, biscuits). Only in the puffs and pouches categories did parents show a significantly higher willingness to buy the baby products over the children's (see Fig. 5). A similar trend emerged when comparing the baby products to adult products in the bars and puffs categories: parents expressed a significantly greater willingness to buy the baby option.

### 3.2 Food regulatory knowledge and its association with willingness to buy

The results in Table 1 emphasize significant gaps in parental knowledge about EU regulations for commercial baby foods. While 72.7 % of respondents were aware that trans fats are prohibited and 69.6 % were aware that EU legislation limits the quantities of salt and sugar in baby foods, knowledge about other regulatory aspects was more inconsistent. In particular, less than half of respondents (45.5 %) correctly recognized that preservatives are not permitted in baby foods. Similarly, only 52.5 % were aware that pesticide regulations for baby foods are stricter than for conventional foods. Although 61.6 % correctly noted that colorants are not allowed, this indicates that nearly 40 % remain unaware of this safety regulation.

The results in Fig. 2 reveal that correlations between parents' knowledge of baby food regulations and their willingness to buy snacks varied across product categories and age groups. For snacks marketed to babies, the correlations were generally statistically significant, for puffs (r = .14, p < .01), pouches (r = .11, p < .01), and crackers (r = .11, p < .01). The correlation was also significant for biscuits (r = .08, p < .05), but not for bars. In contrast, correlations for snacks marketed to children and adults were less consistent. For children's snacks, only bars (r = .08, p < .05) showed a significant correlation, while the remaining categories displayed non-significant or minimal associations. Similarly, for adult snacks, the only significant correlation could be observed for crackers (r = .10, p < .05).

## 4 Discussion

Commercial snacks are increasingly included in the diets of infants and toddlers (Deming et al., 2017; Garcia et al., 2020; Shriver et al., 2018), yet limited research has examined how parents evaluate these products across different target age groups. This study addressed this gap by exploring parental evaluations of commercial snacks marketed for babies, children, and adults. Additionally, we investigated the relationship between parents’ regulatory knowledge and their purchasing intentions for snacks across these age groups. By combining these perspectives, our findings provide insights into parental decision-making processes and highlight areas where regulatory knowledge may influence—or fail to influence—choices for infants and toddlers.

### 4.1 Parents’ perceptions of snacks for babies, children, and adults

Our findings suggest that parents generally perceive the attributes of snacks similarly across products targeted at babies, children, and adults, while a few minor product-specific differences emerged. For instance, some products targeted at babies were slightly rated more positively in terms of healthiness compared to those for children and adults. However, in many cases, parents’ perceptions showed little differentiation across age groups. These results suggest that parents may either struggle to distinguish between products designed for different age groups or perceive few meaningful differences in their attributes. Previous research examining the nutritional quality of biscuits found that baby-specific products generally exhibited higher nutritional quality compared to similar products marketed for older children and adults (Klerks et al., 2023). This highlights that products designed for infants and toddlers may provide additional benefits in terms of nutrition and ingredient composition.

This limited distinction, we found in our study, may stem from gaps in parental knowledge about the stricter regulations of baby-specific products. As discussed earlier in the introduction, these regulations impose more stringent limits on contaminants like pesticides and heavy metals as well as on nutritional components such as sugar and salt (Commission Directive 2006/125/EC; Commission Regulation (EC) No. 609/2013, 2013), offering significant safety and nutritional benefits. However, parents may not be fully aware of these regulatory advantages, which lowers their motivation to choose baby-specific products (which are generally more expensive) over snacks marketed for older children or adults. However, in two out of five product categories, parents showed a slightly greater willingness to purchase baby snack foods, indicating a potential preference for these options.

Interestingly, parents demonstrated a higher willingness to purchase baby-specific products in certain categories, such as puffs and pouches. Research exploring why parents choose packaged foods for their infants revealed that puffs and pouches are popular due to their texture, which minimizes the risk of choking (Hässig-Wegmann et al., 2025; Isaacs et al., 2022). This makes them a safe and convenient snack option for infants and toddlers (Isaacs et al., 2022). Furthermore, due to their texture properties, these product types are almost exclusively marketed for babies, making them more recognizable as baby-specific snacks. In contrast, categories like bars and crackers often overlap with products for older children and adults, reducing their perceived distinctiveness. These findings highlight the influence of visual cues, such as packaging design, on parental decision-making.

### 4.2 Regulatory knowledge and its association with buying intentions

Our analysis of parental knowledge about baby food regulations revealed significant gaps in understanding. While many parents were aware of specific regulations, such as the prohibition of additives and trans fats, fewer demonstrated knowledge of restrictions on colorants, preservatives, and pesticides. These findings align with prior research suggesting limited awareness of EU regulations (Boak et al., 2016; Hässig-Wegmann et al., 2024).

Parents' knowledge of baby food regulations was more consistently associated with their willingness to buy snacks marketed for babies. However, the correlations were relatively low, indicating that knowledge alone does not significantly drive purchasing intentions. This was particularly evident in the case of parents' willingness to buy baby-specific bars, which showed no significant correlation with regulatory knowledge. This outcome may be attributed to competing factors, such as the strong visual appeal of child-specific packaging. For instance, the child-specific bar prominently featured vibrant imagery of strawberries, which might have conveyed a sense of healthiness and freshness, while the baby-specific bar, with its simpler design featuring a single banana, may have been perceived as less attractive or nutritious. Previous research highlights the impact of packaging on product perception and purchasing decisions (Mehta et al., 2012; Tang et al., 2020). When combined with the findings from our study, it appears that these visual cues often outweigh the influence of regulatory knowledge, illustrating how heuristic factors can dominate decision-making in this context.

The low correlations between regulatory knowledge and willingness to buy highlight a critical knowledge-behavior gap. One possible explanation, grounded in the Elaboration Likelihood Model (ELM) (Petty and Cacioppo, 1986), is that decisions about baby snacks are more likely to follow the peripheral route of processing. Parents may rely on heuristic cues such as packaging design, brand familiarity, or visual appeal rather than engaging in detailed, central-route evaluations of regulatory compliance. Baby snacks are often seen as supplemental rather than essential components of a child's diet, reducing the likelihood of deliberate, regulation-driven decision-making (Isaacs et al., 2022; Killion et al., 2023). Furthermore, marketing strategies may exacerbate this gap. Research indicates that certain packaging claims influence parents' product perceptions and their purchase intentions, possibly purchasing unhealthy snacks for their infants (Dixon et al., 2024; García et al., 2019; McCann et al., 2022). Such claims can mislead parents, undermining the perceived benefits of regulated baby snacks.

### 4.3 Practical implications

Our findings raise important concerns about whether parents can reliably differentiate baby-specific products from snacks marketed for older children or adults. While baby snacks are subject to stricter safety and nutritional regulations, including limits on contaminants like pesticides and heavy metals, parents showed limited awareness of these protections. For parents who were more informed, this knowledge did not consistently translate into higher intentions to purchase baby-specific snacks, suggesting they may also choose other options, such as homemade snacks. Nevertheless, this pattern highlights a gap between knowledge and behavior, underscoring the need for clearer communication and strategies to guide parents toward better snack choices for their infants and toddlers.

The recently introduced WHO Nutrient and Promotion Profile Model (NPPM) (World Health Organization, 2022) for infants and young children (6–36 months) further tightens restrictions on ingredients such as added sugars, salt, and fat, offering stronger protections for young children's health. However, the NPPM also restricts the use of claims like "no added sugar" or "rich in vitamin C," which are currently allowed under EU regulations. While this policy aims to prevent misleading claims, it may unintentionally make it more difficult for parents to identify healthier baby-specific options. For instance, snacks made entirely of fruit might no longer be distinguishable from those with added sugar, potentially leading parents to choose less suitable products. Policymakers should consider allowing transparent and truthful claims that help parents recognize the advantages of baby-specific snacks while ensuring they are not misled by exaggerated marketing.

The inability to clearly differentiate baby-specific snacks from those for older children or adults is particularly concerning given prior findings that baby snacks are often healthier and more natural than their counterparts (Klerks et al., 2023). Without clear distinctions, parents may choose snacks for older children or adults based on factors like cost or convenience, inadvertently exposing their infants to products with lower nutritional value or higher levels of contaminants (Roman and Sanchez-Siles, 2025).

Ultimately, tackling these challenges requires a coordinated approach. This includes improved labeling, aligned regulations, and enhanced parental education. Infants and toddlers are especially vulnerable during their first 1000 days of life, a critical period for establishing lifelong healthy eating habits and preventing health issues such as obesity (Koletzko et al., 2017). Ensuring that young children are provided with safe, nutritionally adequate snacks is essential for supporting their growth and development. By empowering parents with the right tools and options, policymakers and manufacturers can help build a healthier future for the youngest generation.

## 5 Limitations and future research

Like any other empirical study, this research has several limitations that can guide future research. First, the study relied on an online survey conducted in Germany, which, while representative of the German population in terms of gender and educational distribution, may limit the generalizability of findings to other cultural or regulatory contexts. Future studies could expand to include diverse countries with varying food regulations to explore whether similar patterns emerge globally. Second, the study assessed parents' perceptions based on images of product packaging, which excluded ingredient lists and nutritional information. While this approach aligns with real-world scenarios where visual and marketing cues heavily influence decisions, future research should examine the impact of providing explicit regulatory or nutritional information alongside product imagery on parental evaluations and purchasing intentions. Third, the food regulatory knowledge scale used to examine correlations with participants' willingness to buy had certain limitations. The scale indicated that parents had a moderate to high level of knowledge about infant food regulations. While this suggests that participants were relatively well-informed, such high levels of knowledge could have introduced bias into our analysis. Additionally, the scale's Cronbach's alpha was only moderate (.63), and as a consequence, the relationship between participants' knowledge and their willingness to buy has been underestimated in our findings. Furthermore, we did not ask participants whether they include commercial snacks in their infants' diets or consume them personally. While this approach allowed us to capture a more diverse and potentially representative sample of the general parent population—including those without prior exposure to commercial snacks—this lack of previous experience may have influenced participants' evaluations. In particular, it is possible that some of the lowest ratings observed (e.g., scores of 0 in the boxplots) came from parents who are unfamiliar with or actively avoid commercial snacks. Future research could address this by assessing whether parents incorporate commercial snacks into their infants' diets, as well as their personal consumption and perceptions of snacks, to gain deeper insights into the factors shaping perceptions and buying intentions for commercial snacks. Lastly, price was not included in our study design, as our primary focus was to assess the extent to which parents were able to differentiate snacks marketed to different age groups. However, since baby snacks are often more expensive than those aimed at older children or adults, future research could explore how price affects parents' perceptions of snack attributes across different age groups.

## 6 Conclusions

This study sheds light on the complex factors shaping parents' perceptions of commercial snacks marketed for babies, children, and adults, as well as the influence of regulatory knowledge on purchasing intentions. Parents generally found it difficult to identify significant differences in snack attributes across products targeted at different age groups. This lack of consistent differentiation between baby snacks and those for older age groups underscores a significant limitation in parents’ ability to recognize and act on the stricter safety and nutritional standards of baby-specific products. In addition, the correlations between regulatory knowledge and willingness to buy baby-specific snacks were generally weak.

Our findings highlight the critical need for interventions. Improved labeling and stricter regulation of health-related claims are essential to ensure parents are equipped to make informed choices. Clear communication of the benefits of baby-specific products, alongside collaborative efforts to maintain their affordability and convenience, can support healthier snack choices for infants and toddlers. By addressing these issues, policymakers and industry stakeholders can help foster better-aligned parental decisions during the critical early years of childhood development.

## Funding sources

This work has been jointly funded by the Consumer Behavior Group at 10.13039/501100003006ETH Zurich and Hero Group Lenzburg Switzerland.

## Declaration of competing interest

Author Luisma Sanchez-Siles works for the Hero Group Lenzburg Switzerland.

## Figures and Tables

**Table:** 

Target age	Baby	Child	Adult
Product category			
Bar	Organic bar made with blueberries, banana, and oats. Sweetened with fruit. (Green bubble: High in fiber and 6-pack.) (Red Heart: from 12 months onwards)	Fruity oat bar with strawberries, and oats. Sweetened only with fruit. (Green bubble: With whole grain)	Oat bar with blueberries.
Cracker	Organic Spelt-Oat Rings. Without added sugar and salt. From 8 months onwards.	Organic Pretzel Seahorses with spelt. For the lunch box. Without spreading salt. Without palm oil. With 47 % spelt.	Spelt Prezel with sesame.
Puff	Crispy snack with carrot and pumpkin. From 8 months onwards. With organic corn. Without added sugar.	Organic spelt balls with tomato and carrot. Vegan. With 51 % spelt.	Carrot cracker. With spelt.
Pouch	Peach, apricot, banana with spelt. 98 % fruit. From 8 months onwards. Climate positive. Without added sugar.	Apple, raspberry, yoghurt with spelt and oat.	Fruit Snack with apple, banana and strawberries. From certified organic farming. 100 % fruit. Without added sugar.
Biscuit	Spelt biscuits. From 10 months onwards.	Butter biscuits. Traditional recipe. Sweetened with an alternative. Made with real butter and bourbon vanilla.	Spelt butter biscuit.

**Table:** 

Product	Target age	Snack category	Mean (SD) Willingness to buy	Mean (SD) Appropriateness	Mean (SD) Texture	Mean (SD) Healthiness	Mean (SD) Naturalness
	Baby	Puff	66.26 (29.02)	65.90 (25.86)	75.30 (26.08)	61.00 (27.09)	61.03 (28.31)
	Child	Cracker	65.85 (29.09)	63.14 (26.64)	70.77 (27.42)	57.62 (27.21)	62.16 (27.76)
	Baby	Cracker	63.06 (29.74)	65.90 (25.58)	74.36 (26.45)	61.74 (26.49)	57.77 (28.16)
	Baby	Pouch	62.17 (32.85)	62.27 (28.82)	73.08 (29.32)	56.10 (30.07)	57.88 (30.84)
	Adult	Pouch	60.77 (32.79)	61.86 (29.06)	72.54 (29.14)	56.51 (30.44)	60.41 (29.95)
	Adult	Cracker	60.51 (31.26)	59.27 (28.30)	66.39 (29.78)	54.66 (29.18)	55.73 (29.88)
	Child	Bar	60.36 (29.93)	62.90 (26.58)	68.20 (27.52)	59.40 (27.54)	58.70 (28.81)
	Baby	Bar	60.33 (31.21)	62.94 (25.90)	67.83 (27.90)	59.10 (27.33)	58.82 (27.81)
	Adult	Biscuit	58.77 (31.33)	55.91 (29.30)	72.62 (27.30)	51.25 (30.80)	52.06 (30.64)
	Adult	Puff	58.19 (30.09)	63.76 (26.13)	67.58 (27.69)	60.79 (27.47)	60.20 (28.49)
	Baby	Biscuits	58.07 (29.55)	60.75 (26.74)	71.64 (26.60)	55.49 (28.32)	55.58 (29.10)
	Child	Biscuit	57.42 (31.23)	56.72 (29.27)	71.75 (27.03)	51.47 (30.15)	52.73 (30.14)
	Child	Puff	56.43 (31.40)	62.15 (26.13)	65.00 (28.52)	59.29 (26.58)	57.77 (28.16)
	Adult	Bar	52.15 (31.61)	60.33 (27.08)	60.00 (30.00)	58.47 (27.86)	58.11 (27.83)
	Child	Pouch	48.29 (35.70)	48.75 (31.69)	70.08 (30.92)	43.10 (32.24)	42.66 (32.76)

## Data Availability

Data will be made available on request.

## Footnotes

1 Similar to Klerks et al. (2023) and for the sake of brevity, we use "babies" or "infants and toddlers" interchangeably throughout this article, with both terms referring to children younger than 3 years.

2 Extrusion is a production method in which raw materials, typically cereals, grains, or starches, are subjected to high temperature and pressure to expand and form specific textures, such as puffed, crispy, or crunchy.

3 Education levels: low (none, compulsory school, social year), middle (apprentice ship, middle school), high (university of applied sciences or university).
